# Improving quality of common reed (*Phragmites communis* Trin.) silage with additives

**DOI:** 10.5713/ajas.17.0807

**Published:** 2018-04-12

**Authors:** Keigo Asano, Takahiro Ishikawa, Ayako Araie, Motohiko Ishida

**Affiliations:** 1 Department of Bioproduction Science, Faculty of Bioresouces and Environmental Science, Ishikawa Prefectural University, Ishikawa 921-8824, Japan

**Keywords:** Water-soluble Carbohydrate, Lactic Acid Bacteria, Cellulase, Harvest Times, Nitrogen Fertilization

## Abstract

**Objective:**

Common reed (*Phragmites communis* Trin.) could potentially provide an alternative resource for silage; however, its silage quality is poor. The aim of this study was to investigate the factors in reed that contribute to poor quality and determine how the use of additives at ensiling could improve fermentation quality.

**Methods:**

In Experiment 1, we determined the chemical composition and the presence of indigenous lactic acid bacteria (LAB) in reed. We further examined fermentation quality of reed silage under conditions without additives (NA) and treated glucose (G), lactic acid bacteria (L), and their combination (G+L). In Experiment 2, silage of NA, and with an addition of cellulase and lactic acid bacteria (CL) were prepared from harvested reed. The harvested reeds were fertilized at nitrogen concentrations of 0, 4, 8, and 12 g N/m^2^ and were harvested thrice within one year.

**Results:**

The indigenous LAB and fermentable carbohydrates are at extremely low concentrations in reed. Reed silage, to which we added G+L, provided the highest quality silage among treatments in Experiment 1. In Experiment 2, N fertilization had no negative effect on silage quality of reed. The harvest times decreased fermentable carbohydrate content in reed. The CL treatment provided a higher lactic acid content compared to the NA treatment. However, the quality of CL treated silage at the second and third harvests was significantly lower than at the first harvest, due to a reduction in carbohydrates caused by frequent harvesting.

**Conclusion:**

The causes of poor quality in reed silage are its lack of indigenous LAB and fermentable carbohydrates and its high moisture content. In addition, reed managed by frequent harvesting reduces carbohydrate content. Although the silage quality could be improved by adding CL, higher-quality silage could be prepared by adding fermentable carbohydrates, such as glucose (rather than adding cellulases).

## INTRODUCTION

According to the Ministry of Agriculture, Forestry and Fisheries, based on total digestible nutrients, feed self-sufficiency in Japan was only 27% in 2015 [1]. Increasing domestic feed production is a critical issue in Japan. The common reed (*Phragmites communis* Trin.) has been studied recently as a potential feed or bioenergy resource (due to its ability to produce high-yields of dry matter [DM] [2,3]) and for its potential for providing roughage for ruminants [4,5]. Furthermore, common reed is distributed widely in abandoned paddy fields and at riverside sites throughout Japan, most of them is burned or left useless, and so its utilization as animal feed could potentially improve Japan’s feed self-sufficiency. The primary method of preserving forage is via hay or silage. Silage is more suitable in Japan, where the weather is rainy and humid in spring and summer (when reed is harvested) [6]. However, the quality of fermented reed silage has been shown to be poor and not optimal for storage and feeding (its pH is >4.3 [7]). Its quality would also have to be improved before it could be used as roughage.

Usually, the poor quality of silage is due to its inherent mois ture, fermentable carbohydrate content, and the presence of lactic acid bacteria (LAB) [8,9]. In addition, harvest time within a year and N fertilization rate may affect silage quality by changing chemical composition in grasses [6,10]. However, to the best of our knowledge, there is limited information on the factors causing the poor quality of reed silage. Silage additives are widely used for improving silage fermentation, which include LAB, a substrate such as molasses for LAB growth, and cellulases. The use of cellulase at ensiling has been used to enhance fiber degradation and produce substrates, such as water-soluble carbohydrates (WSC) [11]. Therefore, the use of LAB, WSC, and cellulase at ensiling are expected to improve the fermentation quality of common reed silage.

In this study, we conducted two experiments to investigate the factors responsible for poor quality of reed silage and investigate potentially useful additives for improving its quality. The aims of Experiment 1 were to identify the indigenous populations of LAB in common reed, quantify reed’s WSC content, and compare the quality of fermented silage by adding LAB or/and glucose to the silage without additives. The aims of Experiment 2 were to investigate the effects of various reed-management strategies on fermented reed quality, including harvest times, varying N fertilization rates, and the addition of LAB and cellulase at ensiling.

## MATERIALS AND METHODS

### Experimental site and reed management

#### Experiment 1

Reed was collected from an abandoned paddy field (10 m×10 m) located on reclaimed land in Kahokugata, Kanazawa, Ishikawa Prefecture (36°40′ N, 136°41′ E). On 20 April 2010, all plants and withered residues were cut using a bush cutter and removed from the field. On 20 May, spring-grown reed was cut at a height of approximately 10 cm above the ground using hand sickles and collected. The collected reed was finely chopped with a hay cutter (22-mm cutting length) for silage preparation and then subjected to indigenous LAB analysis. Portions of the chopped grass were oven dried at 60°C for 24 h for chemical composition analyses. On 3 August, summer-grown reed was harvested (using the same methods as above) after cutting and removing all plants on 25 June. Indigenous LAB populations were analyzed from the summer-grown reed.

#### Experiment 2

Reed was collected from a dry riverbed in Kahokugata (36°49′ N, 136°40′ E). The collection field, dominated by reed, had been managed for about 40 years by cutting the reed once per year to keep the river channels clear. Twelve plots (3 m×3 m) were established in an experimental area (17 m×22 m) of the field on 19 April 2014, after cutting and removing all plants growing in the area. Four N fertilization treatments (with three replicates per treatment) were randomly assigned to the 9-m^2^ plots: 0 g N/m^2^ (0N), 4 g N/m^2^ (4N), 8 g N/m^2^ (8N), and 12 g N/m^2^ (12N). N fertilizer (14% N, 14% P_2_O_5_, and 14% K_2_O) was top-dressed (surface spread) in each plot after all plants had been removed. Reed was harvested three times (25 May, 27 June, and 31 July 2014) from 1 m×1 m subplots within each 9-m2 plot. Reed was harvested using sickles, at a height of approximately 10 cm above ground. At each harvest, all plants in the plots (and experimental area) were cut using a bush cutter and removed from the area before adding N fertilizer. The collected reed was finely chopped with a hay cutter for silage preparation. Portions of the chopped grass were oven dried for chemical composition analyses.

### LAB analysis

The common reed collected in Experiment 1 (20 May and 3 August 2010) was immediately classified into the following three groups based on height: ≤0.5 m (short-statured), 0.5 to 1.0 m (mid-statured), and ≥1.0 m (tall-statured). Reed in each group was cut using sterilized scissors to a length of 1 cm for indigenous LAB analysis. A 10 g portion of the cut reed was blended with 90 mL of sterilized distilled water. Serial dilutions were plated on de Man, Rogosa and Sharpe (MRS) agar plates (Difco Laboratories, Detroit, MI, USA). The agar plates were then incubated in an anaerobic vessel at 30°C for 48 h. The grown colonies were grouped by their shapes and colors and counted as colony forming units (CFUs). Representative colonies were cultivated in 4 mL of MRS medium at 30°C overnight and used for species identification. Genomic DNA was extracted, according to the manufacturer’s instructions, using the Wizard Genomic DNA Extraction Kit (Promega, Fitchburg, MA, USA). Whole regions of the 16S ribosomal RNA gene (rDNA) were amplified with Ex Taq DNA Polymerase (Takarabio, Shiga, Japan) using F7 (5′-AGAGTTTGA TYMTGGCTCAG-3′) and R1510 (5′-ACGGYTACCTTGT TACGACTT-3′) as polymerase chain reaction (PCR) primers. The amplified fragments were purified using the QIAquick PCR Purification Kit (Qiagen, Venlo, Netherlands). The PCR protocol was as follows: 96°C for 2 min, followed by 25 cycles of denaturation at 96°C for 15 s, primer annealing at 50°C for 15 s, and extension at 72°C for 1.5 min. The sequencing reaction was performed using the BigDye XTerminator Purification Kit (Applied Biosystems, Carlsbad, CA, USA) and DNA sequences were analyzed using the 3130xl Genetic Analyzer (Applied Biosystems, USA). Nucleotide sequences of multiple, hyper-variable regions of 16S rDNA genes were analyzed and bacterial species were identified according to the GenBank database using the BLAST program at the National Center for Biotechnology Information.

### Silage preparation

#### Experiment 1

Four silage treatments were prepared as follows: no-additives (NA treatment), 2% glucose on a fresh matter (FM) basis (G treatment), 0.0017% LAB (*Lactobacillus plantarum* Chikuso-1; Snow Bland Seed Co., Hokkaido, Japan) on an FM basis (L treatment), and glucose plus LAB addition (G+L treatment). A glucose solution (1 g/mL distilled water) was prepared by heating it at 40°C. Reed (100 g FM) of the G, L, and G+L treatments were mixed with a 2 mL glucose solution and 1 mL distilled water, a 1 mL of LAB solution (0.0017 g/mL distilled water) and 2 mL distilled water, and a 2 mL glucose solution and 1 mL of LAB solution, respectively. The volume of water addition in the NA treatment was an equivalent amount (3 mL) of distilled water. Then, 100 g of each of these mixtures was packed into plastic pouches (Hiryu KN; Asahi Kasei, Tokyo, Japan) and vacuum-sealed. Five replicate pouches were prepared for each treatment; all the pouches were stored for two months at room temperature (18°C to 22°C).

#### Experiment 2

Two silage treatments for each N treatment were prepared as follow: no-additives (NA treatment) and 0.0017% commercial additive (Si Master AC; Snow Bland Seed Co., Hokkaido, Japan) that contained acremonium cellulase and LAB (*Lactobacillus paracasei* and *Lactococcus lactis*) on a FM basis (CL treatment). The additive solution (6.8% commercial additive) was added to the reed in the CL treatment. Reed of the NA treatment were supplemented with the same amount of distilled water as that used for the CL treatment. Then, 100 g of each mixture was packed into plastic pouches and sealed. Six pouches (three replicate pouches from NA and CL, respectively) were prepared from reed in each experimental plot. All pouches were stored for two months at room temperature (18°C to 22°C).

### Chemical analyses

The DM, crude ash (CA), and ether extract (EE) were determined following the method described in Abe [12]. Crude protein (CP) was determined with a nitrogen and carbon analyzer (Sumigraph Model NL-220F; Sumika Chemical Analysis Service, Tokyo, Japan). The organic cellular contents (OCC), nitrogen-cell wall free extract (NCWFE), organic cell wall (OCW), organic a (Oa; high-digestible fraction in OCW), and organic b (Ob; low-digestible fraction in OCW) were determined by a feed analysis based on the enzymatic method [13,14].

The pH and organic acids of silage were determined using the method described by Cai [15] with slight modifications. Silage was cut to a length of approximately 5 mm using scissors, and then 50 g of the cut silage was mixed with 140 mL of distilled water and then stored for 24 h at 4°C. After 24 h, the mixture was filtered through four layers of gauze. The pH of the filtrate was determined using a glass-electrode pH meter (F-52; Horiba, Tokyo Japan). The filtrate was centrifuged at 1,600×g for 15 min and the supernatant was used for the analysis of ammonia-nitrogen (NH_3_-N). The NH_3_-N content was determined according to the indophenol method [16]. The supernatant was filtered using a disposable membrane filter (DISMIC 13CP045AN; ADVANTEC, Tokyo, Japan) and the filtrate was used to determine organic acid content. The lactic acid, acetic acid, propionic acid, and butyric acid contents in the filtrate were determined using high performance liquid chromatography (Prominence Organic Acid Analysis System; Shimadzu, Kyoto, Japan).

### Statistical analyses

All statistical analyses were performed using SPSS 18 for Windows (SPSS Japan Inc., Tokyo, Japan). Differences in the fermentative parameters of reed silage in Experiment 1 were evaluated by one-way analysis of variance (ANOVA) using the general linear model procedure and then means were separated based on Tukey’s Honest Significant Difference test. In Experiment 2, the effects of harvest time, N fertilization, and additive use on the chemical composition in reed and quality of fermented reed silage were evaluated with a repeated measure ANOVA. Means were separated based on the Bonferroni procedure. The association between variables indicating the quality of fermented reed silage and chemical composition in reed was evaluated with correlation analyses. All analyses used p<0.05 as a criterion of statistical significance.

## RESULTS

### Experiment 1

Two LAB species, *Carnobacterium maltaromaticum* (*C. maltaromaticum*) and *Enterococcus sulfurous* (*E. sulfurous*), were identified from the isolated colonies (Table 1); the other species identified were non-LAB species (*Staphylococcus* spp., *Listeria* spp., and *Bacillus* spp.). No LAB species were detected from the mid- and tall-statured reeds harvested in either May or August (spring-grown reed or summer-grown reed). The concentration of *C. maltaromaticum* in small-statured reed harvested in May was 2.26 log CFU/g FM. The concentration of *E. sulfureus* in small-statured reed harvested in August was 2.00 log CFU/g FM. In our experiment, the duration of reed growth harvested for ensilage was 31 days. The WSC content was 53 g/kg DM (Table 2) and the values on a DM and FM basis were 5.3% and 0.9%, respectively.

The pH, acetic acid, and NH _3_-N concentrations in the NA treatment were the highest among all the treatments, while lactic acid content was very low (Table 3). The pH, lactic acid, acetic acid, and butyric acid concentrations did not significantly differ between the NA and L treatments (p≥0.05). The pH, acetic acid, propionic acid, and NH_3_-N concentrations were significantly lower, while the lactic acid concentration was significantly higher in the G treatment than in the L and NA treatments (p<0.05). The pH, acetic acid, butyric acid, and NH_3_-N concentrations were significantly lower, while the lactic acid concentration was significantly higher in the G+L treatment than in the NA, G, and L treatments (p<0.05). The pH and lactic acid content in the G+L treatment were 3.90 and 14.19 g/kg FM, respectively.

### Experiment 2

The duration of pre-harvest growth for reed were 35, 34, and 35 days at the first, second, and third harvests, respectively. Except for CP, the chemical composition of reed was not affected by N fertilization (p≥0.05; Table 4). However, CP content was significantly increased by N fertilization (p<0.01). CP content in the 8N and 12N treatments was significantly higher than it was in the 0N and 4N treatments (p<0.05) (Table 5). Except for Oa, chemical composition was significantly affected by harvest time within a year (p<0.001; Table 4). Moisture, organic matter (OM), OCC, CP, EE, and NCWFE contents from reed collected at the third harvest were significantly lower than from reed collected at the first and second harvests (p<0.05). Mean CP content at the first, second, and third harvests were 204, 185, and 179 g/kg DM, respectively (Table 5). The mean moisture content at the first, second, and third harvest was 803, 784, and 763 g/kg FM, respectively. The mean NCWFE content at the first, second, and third harvest was 64.46, 49.77, and 16.43 g/kg DM, respectively. OCW and Ob were significantly different among the three harvest times and frequent harvesting significantly increased mean Ob content (p<0.05). Oa content in reed was not affected by either N fertilization or harvest time within a year (p≥0.05). In this experiment, there were no interactions between N fertilization and harvest time relative to the chemical composition of reed (p≥0.05; Table 4).

Moisture, pH, NH _3_-N, lactic acid, acetic acid, and propionic acid of reed silage were not significantly affected by N fertilization (p≥0.05; Table 4). However, butyric acid was significantly reduced following N fertilization (p<0.05). The butyric acid content significantly decreased from 1.44 g/kg in the 0N treatment to 0.78 g/kg FM in the 12N treatment (p<0.05; Table 6). Moisture and pH of the silage were related to both harvest time of reed within a year and use (at ensiling) of the additive containing cellulase and LAB (p<0.05; Table 4). The mean moisture content decreased by frequent harvesting (p<0.05; Table 7). The mean moisture content of the NA and CL treatments was 772 and 756 g/kg FM, respectively, which decreased with the use of the additive (p<0.05). The pH of the silage increased with increasing frequency of harvest (p<0.05). The pH of the NA and CL treatments decreased with the use of the additive (p<0.05). Propionic acid was significantly affected by both harvest time within a year and use of the additive (p<0.01; Table 7).

**Figure 1:**
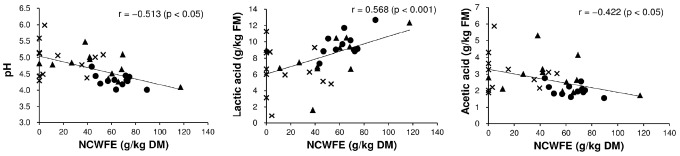
Relationship between the nitrogen cell wall-free extract (NCWFE) content of common reed and fermentation quality of the silage to which cellulase and lactic acid bacteria were added (the CL treatment). ●, first harvest; ▲, second harvest; ×, third harvest.

Lactic acid in the silage was significantly affected by the interaction of harvest time and use of the additive (p<0.001; Table 4). Lactic acid content of the CL treatment was lower at the third harvest relative to the first harvest (p<0.05; Table 7). In contrast, lactic acid content in the NA treatment was higher at the third (and second) harvests relative to the first harvest (p<0.05). Mean lactic acid content increased with the use of the additive (p<0.05): lactic acid content was 4.02 and 8.07 g/kg FM in the NA and CL treatments, respectively. Acetic acid, butyric acid, and NH_3_-N were significantly related to the interaction of harvest time and use of the additive (p<0.05; Table 4). Use of the additive was significantly and negatively related to the mean contents of acetic acid (5.64 to 2.69 g/kg FM), butyric acid (2.04 to 0.31 g/kg FM), and NH_3_-N (1.12 to 0.54 g/kg FM) (p<0.05; Table 7). Acetic acid and NH_3_-N contents in the CL treatment were higher at the second and/or third harvest relative to the first harvest (p<0.05). The acetic acid of the NA treatment did not significantly differ among the three harvests (p≥0.05). The butyric acid and NH_3_-N contents of the NA treatment were lower following more frequent harvesting (p<0.05). In the CL treatment, pH, lactic acid, and acetic acid of silage were correlated with NCWFE in reed (Figure 1). Negative correlations between the NCWFE and pH (r = −0.513; p<0.05) and acetic acid (r = −0.422; p<0.05) were detected; in contrast, a positive correlation between the NCWFE and lactic acid was detected (r = 0.568; p<0.001). Other chemical components in reed did not significantly correlate with the quality of the reed silage (p≥0.05).

## DISCUSSION

There have been no studies indicating that *C. maltaromaticum* is useful for preparing silage and it has been reported that the species is a spoilage bacteria that decomposes food products, such as meat and fish [17]. Some *Lactobacillus* and *Lactococcus* species are effective as LAB for improving the fermentation quality of silage due to their high lactic acid productivity and their tolerance to acidic conditions [18]. Li and Nishino [19] reported that *E. sulfureus* was eradicated in guinea grass silage when pH declined from 6.12 to 5.22 at day 28 after ensiling, whereas *L. plantarum* increased, even under low pH conditions, and survived until day 120 after ensiling. The preparation of high-quality silage has been shown to be difficult when *E. sulfureus* was the only LAB in the silage. Morichi and Ohyama [20] investigated indigenous LAB populations in forage and reported that *Lactobacillus* species was not detected in approximately one-third of 201 forage samples. They also found that the number of *Lactobacillus* species in forage was higher in summer than in spring and autumn. In Experiment 1of our study, *Lactobacillus* species were not detected in reed in either spring or summer harvests. Therefore, we suspect that LAB, such as *Lactobacillus* species, do not exist in, or rarely inhabit common reed, at any time of the year. Moreover, the concentration of LAB in the material required to prepare high-quality silage has been found to be ≥5 log CFU/g FM [8,18]. However, the concentration of LAB in the common reed we sampled (Experiment 1) was <5 log CFU/g FM. Based on all these studies, we conclude that the species and numbers of LAB normally inhabiting reed are ill-suited for preparing high-quality silage from common reed.

The quality of fermented silage becomes poor when the WSC content of the material falls below 10% of DM or 2% of FM [9,21]. However, the WSC content of reed in our study was only 5.3% of DM and 0.9% of FM. Therefore, the WSC content in our experiment was approximately half the value required for producing good-quality silage. As a result, silage without additives ferments poorly. In general, silage with a pH of ≤4.2 can inhibit the activity of unwanted bacteria and fungi, which in turn hinders decomposition and deterioration, which together longer stabilizes the quality of silage [22]. We found that the quality of fermented reed silage was improved more by adding glucose than by adding LAB at time of ensiling. However, because the pH of the G treatment silage was 4.63, which is too high, addition of glucose alone would not be a practical choice for ensilage. In contrast, the G+L treated silage had a higher lactic acid content, lower pH, and lower acetic acid, butyric acid, and NH_3_-N contents than our other treatments. As a result, it was ideal for fermenting to silage. Therefore, we found that high-quality silage could be prepared from common reed by using two additives: LAB (such as *Lactobacillus* species) and a substrate (such as glucose). However, use of glucose as additive of a substrate not realistic. Because molasses is often added as a substrate additive and improve fermentation quality of silage [23], we propose that molasses used instead of glucose.

In the present study, common reed was harvested at fixed intervals to prevent extreme changes in the chemical composition that occur after growing for more than 40 days [6]. In Experiment 2, the effect of harvest time was observed for almost all the chemical components we measured, whereas the effect of N fertilization was only related to CP in reed. These results correspond with other studies in which the CP content of grass increased following fertilization with N [24,25]. In general, protein is synthesized from N absorbed through the roots and from carbohydrates, such as WSC; therefore, a large supplement of N decreases the amount of WSC in plants due to a stimulation of carbohydrate consumption [25,26]. However, NCWFE, which is the non-structural carbohydrate fraction containing the WSC, was not significantly affected by N fertilization (p = 0.199) in our experiment. In contrast, the effect of harvest time was significant (p<0.001). Common reed stores carbohydrates (produced by photosynthesis) in its rhizomes for regrowth in the following year. Some studies [27,28] have reported that the carbohydrates in the rhizome of common reed decrease from spring to summer, coinciding with the peak vegetation period, and that the bulk density of rhizomes, which indicates the quantity of carbohydrate reserves, decreases when the reed is cut [29]. In our experiment, reed was harvested thrice from May to July and so we assumed that the decline in NCWFE was caused by the consumption of carbohydrates required for frequent plant regrowth. Moreover, the NCWFE content at the first and second harvest among the N fertilization treatments had a tendency to decrease following a high N fertilization rate, relative to the third harvest. The content of the third harvest was significantly low by the 12N fertilization treatment. Under high N fertilization levels, when proteins are synthesized for regrowth, the exhaustion of carbohydrates in reed can occur. A combination of these management strategies (thrice-annual harvesting and high-N fertilization) would have an adverse effect on the sustainable use of the common reed for producing silage.

In preparing silage from Experiment 2, we found that man agement options that use a fertilization rate of 4 to 12 g N/m^2^ not have a negative effect on the quality of fermented reed silage. On other hand, the effects of harvest time and use of an additive on silage quality were significant. The NA (no-additive) treated reed silage led to an increase lactic acid, a decrease in butyric acid, and the maintenance of high concentrations of acetic acid and NH_3_-N following frequent harvesting. These changes were likely caused by inhibiting *Clostridium* activity by decreasing the moisture content [8]. However, the concentration of NH_3_-N remained at high levels for all three harvest times and that is probably why pH values did not fall much (range, 5.71 to 6.33). In addition, acetic acid was at its highest concentration among the organic acids in the NA treatment. High levels of acetic acid in reed silage was observed in silages formed in response to the NA and L treatments of Experiment 1. Such fermentation has often been obtained when preparing silage from tropical grasses [30]. Even the lowest moisture conditions (third harvest) could not inhibit fermenting bacteria (enterobacteria and hetero-type LAB [8]), or prevent fermentation from producing high acetic acid concentrations and high pH in silage. From our analysis of the chemical compositions of our experimental treatments of reed, it was obvious that the fundamental cause for poor fermentation is a shortage of substrates, such as WSC or NCWFE, for lactic acid fermentation.

Fermentation quality of reed silage improved by adding cellulase and LAB, which caused pH, acetic acid, butyric acid, and NH_3_-N to decrease. The first harvest silage was remarkably improved by adding cellulase and LAB. However, the silage quality of the second and third harvest was lower than the first harvest and the quality of CL treatment remarkably deteriorated with frequent harvesting. From our results of correlations between fermented reed quality of the CL treatment and the chemical composition of reed as silage material (analyzed to investigate the reason(s) for the degradation in quality), we found that there was no relationship between low silage quality and high moisture content of harvested reed (p≥0.05). On other hand, we observed a strong correlation between NCWFE and fermentation quality. We intended to supply substrates for LAB fermentation by adding cellulase. However, our results indicated that silage quality of common reed is dependent on inherent carbohydrates even when cellulase is used at ensiling, and that silage quality becomes unstable under low carbohydrate conditions. Therefore, we conclude that the cause for the reduced effectiveness of our cellulase/LAB additive at the second and third harvests was due to a lowering of pH exacerbated by a lack of sufficient carbohydrates immediately after ensiling.

In conclusion, preparing high-quality silage from common reed without using additives was difficult because the composition and number of indigenous LAB in reed were not suitable for maintaining lactic acid fermentation regardless of plant growth stage or season of harvest, and WSC content was so low. Moreover, although N fertilization did not negatively affect the quality of reed silage, increasing the frequency of harvest provided positive effects to the no-additive silage and negative effects to the cellulase/LAB-treated silage. The additive containing cellulase and LAB improved fermentation quality. However, to optimize silage fermentation for high-quality silage, we suggest that the most effective approach would be to add both additives LAB and a substrate to common reed silage.

## Figures and Tables

**Table 1:** Species and numbers of indigenous LAB in the common reed from Experiment 1

Harvested month	Plant size (m)	Identified LAB species	16S rDNA similarity (%)	The number of bacteria (log CFU/g FM)
May	≤0.5	*Carnobacterium maltaromaticum*	100	2.26
	0.5–1.0	ND	-	-
	≥1.0	ND	-	-
August	≤0.5	*Enterococcus sulfureus*	100	2.00
	0.5–1.0	ND	-	-
	≥1.0	ND	-	-

LAB, lactic acid bacteria; CFU, colony forming unit; FM, fresh mater; ND, not detected.

**Table 2:** Chemical composition (g/kg DM) of the common reed from Experiment 1

Items	
Moisture (g/kg FM)	817
Organic matter	886
Organic cellular contents	236
Crude protein	177
Ether extract	42
Nitrogen cell wall-free extract	43
Organic cell wall	651
Organic a fraction	135
Organic b fraction	516
Water-soluble carbohydrate	53
Glucose	11
Sucrose	2
Fructose	40

DM, dry matter; FM, fresh matter.

**Table 3:** Fermentation quality (g/kg FM) of treated and non-treated reed silage from Experiment 1

Items	NA	G1)	L1)	G+L1)	SEM	p-value
Moisture (g/kg FM)	825^a^	809^bc^	819^ab^	799^c^	2.67	<0.001
pH	5.16^a^	4.63^b^	5.13^a^	3.90^c^	0.12	<0.001
Lactic acid	0.30^c^	6.25^b^	0.44^c^	14.19^a^	1.33	<0.001
Acetic acid	10.08^a^	5.28^b^	9.38^a^	2.26^c^	0.77	<0.001
Propionic acid	4.03^a^	0.85^c^	2.68^b^	0.36^c^	0.34	<0.001
Butyric acid	3.93^a^	2.94^a^	4.44^a^	0.03^b^	0.48	0.001
NH_3_-N	2.33^a^	1.02^c^	2.05^b^	0.35^d^	0.18	<0.001

FM, fresh matter; NA, no-additives treatment; SEM, standard error of the mean.

1) G, L, and G+L indicate the treatments applied glucose, lactic acid bacteria, and glucose plus lactic acid bacteria, respectively.

a–c Means with different superscripts in the same row indicate significant differences (p<0.05).

**Table 4:** Significance (p-value) of main effects and interactions of N fertilization, harvest time within a year, and use of an additive at ensiling in Experiment 2

Items	Effect			Interaction			
	
	N	H	A	N×H	N×A	H×A	N×H×A
p-value							
Chemical composition							
Moisture	0.550	<0.001	-	0.321	-	-	-
OM	0.083	<0.001	-	0.420	-	-	-
OCC	0.238	<0.001	-	0.548	-	-	-
CP	0.002	<0.001	-	0.179	-	-	-
EE	0.055	<0.001	-	0.117	-	-	-
NCWFE	0.199	<0.001	-	0.155	-	-	-
OCW	0.367	<0.001	-	0.556	-	-	-
Oa	0.359	0.646	-	0.615	-	-	-
Ob	0.474	<0.001	-	0.885	-	-	-
Fermentation quality							
Moisture	0.911	<0.001	0.037	0.098	0.979	0.282	0.836
pH	0.464	<0.001	<0.001	0.250	0.811	0.981	0.505
Lactic acid	0.738	0.807	<0.001	0.174	0.911	<0.001	0.072
Acetic acid	0.317	0.065	<0.001	0.012	0.714	0.008	0.163
Propionic acid	0.146	<0.001	<0.001	0.314	0.299	0.111	0.221
Butyric acid	0.040	<0.001	<0.001	0.628	0.283	<0.001	0.796
NH_3_-N	0.814	0.043	<0.001	0.393	0.892	0.002	0.717

N, nitrogen fertilization; H, harvest time within a year; A, use of the additive containing cellulase and lactic acid bacteria; OM, organic matter; OCC, organic cellular contents; CP, crude protein; EE, ether extract; NCWFE, nitrogen cell wall free extracts; OCW, organic cell wall; Oa, organic a; Ob, organic b.

**Table 5:** Chemical composition (g/kg DM) of the common reed from Experiment 2 subjected to N fertilization and frequent harvesting

Items	0 g N/m^2^			4 g N/m^2^			8 g N/m^2^			12 g N/m^2^			SEM
			
	First	Second	Third	First	Second	Third	First	Second	Third	First	Second	Third	
Moisture (g/kg FM)	800	771	752	799	786	771	804	788	757	809	791	766	3.65
OM	894	897	885	886	887	873	885	877	867	885	882	872	1.75
OCC	265	250	200	257	206	185	264	231	188	275	250	192	6.19
CP	201	172	159	193	174	163	207	200	198	213	195	197	3.23
EE	26	28	26	27	26	23	27	30	26	28	29	26	0.32
NCWFE	67.21	76.08	40.42	65.84	32.67	24.26	59.93	35.44	1.01	64.84	54.89	0.01	5.02
OCW	629	648	686	628	680	688	621	647	679	610	632	681	5.60
Oa	93	75	98	99	108	94	100	99	98	88	80	90	2.89
Ob	536	572	588	529	572	595	521	548	581	522	552	591	5.26

DM, dry matter; SEM, standard error of the mean; FM, fresh matter; OM, organic matter; OCC, organic cellular contents; CP, crude protein; EE, ether extract; NCWFE, nitrogen cell wall free extracts; OCW, organic cell wall; Oa, organic a; Ob, organic b.

**Table 6:** Fermentation quality (g/kg FM) of common reed silage in Experiment 2 following N fertilizer application at four rates

Items	N fertilization level (g N/m^2^)				SEM

	0	4	8	12	
Moisture (g/kg FM)	760	763	765	767	3.24
pH	5.29	5.42	5.48	5.25	0.10
Lactic acid	6.52	5.72	5.53	6.42	0.40
Acetic acid	4.27	4.62	4.17	3.60	0.22
Propionic acid	0.54	0.52	0.49	0.43	0.03
Butyric acid	1.44^a^	1.37^ab^	1.12^ab^	0.78^b^	0.19
NH_3_-N	0.85	0.86	0.84	0.78	0.04

FM, fresh matter; SEM, standard error of the mean.

ab Means with different superscripts in the same row indicate significant differences (p<0.05).

**Table 7:** Fermentation quality (g/kg FM) of common reed silage at the first, second, and third harvests from Experiment 2

Items	NA			Mean	SEM	CL			Mean	SEM
	
	First	Second	Third			First	Second	Third		
Moisture (g/kg FM)	802^a^	764^b^	749^b^	772	4.60	781^a^	751^ab^	736^b^	756	4.23
pH	5.71^b^	6.12^a^	6.33^a^	6.05	0.08	4.31^b^	4.75^a^	4.95^a^	4.67	0.08
Lactic acid	2.04^b^	4.28^a^	5.74^a^	4.02	0.46	9.67^a^	8.13^ab^	6.42^b^	8.07	0.45
Acetic acid	5.82	5.31	5.78	5.64	0.22	2.06^b^	2.90^b^	3.13^a^	2.69	0.17
Propionic acid	0.85^a^	0.57^b^	0.57^b^	0.66	0.04	0.41	0.29	0.28	0.32	0.02
Butyric acid	4.39^a^	0.86^b^	0.88^b^	2.04	0.31	0.10	0.37	0.45	0.31	0.10
NH_3_-N	1.25^a^	1.10^ab^	1.01^b^	1.12	0.44	0.42^b^	0.71^a^	0.50^b^	0.54	0.34

FM, fresh matter; NA, no-additives treatment; CL, treatment applied cellulase and lactic acid bacteria; SEM, standard error of the mean.

ab Means within the same silage treatment in the same row with different letters denote a significant difference (p<0.05).
